# A Wavelet-Based Multiresolution Reconstruction Method for Fluorescent Molecular Tomography

**DOI:** 10.1155/2009/294545

**Published:** 2009-07-22

**Authors:** Wei Zou, Jiajun Wang, Kongpei Wu, David Dagan Feng

**Affiliations:** ^1^School of Electronics and Information Engineering, Soochow University, Suzhou 215021, China; ^2^Department of Electronic and Information Engineering, Hong Kong Polytechnic University, Hong Kong; ^3^School of Information Technologies, University of Sydney, Sydney, NSW 2006, Australia; ^4^Med-X Research Institute, Shanghai Jiao Tong University, Shanghai 200030, China

## Abstract

Image reconstruction of fluorescent molecular tomography (FMT) often involves repeatedly solving large-dimensional matrix equations, which are computationally expensive, especially for the case where there are large deviations in the optical properties between the target and the reference medium. In this paper, a wavelet-based multiresolution reconstruction approach is proposed for the FMT reconstruction in combination with a parallel forward computing strategy, in which both the forward and the inverse problems of FMT are solved in the wavelet domain. Simulation results demonstrate that the proposed approach can significantly speed up the reconstruction process and improve the image quality of FMT.

## 1. Introduction

The use of near-infrared (NIR) light in biomedical research has made significant progress over the past few years [1]. It has been shown that light with wavelengths in the near-infrared range can propagate through tissue for distances on the order of multiple centimeters, because of low tissue absorption in the “near-infrared window.” This finding has encouraged the development of fluorescence techniques to visualize specific biochemical events inside living subjects (in vivo molecular imaging) [2]. Fluorescence techniques have played a critical role in the description of biological processes at the molecular and cellular levels [3]. One particular example is fluorescent molecular tomography (FMT), which is an emerging tool for molecularly based medical imaging [1]. In this imaging modality, a fluorescent biochemical marker used as contrast agent is injected into the biological system and consequently accumulates in diseased tissue as a result of the increased vascular density or by means of selective targeting [4]. During the imaging process, light at the fluorophore's excitation wavelength is launched into the tissue, and then it is absorbed by fluorophore that presents in the tissue, and the fluorophore is elevated to an excited state and remains there for some period of time (the fluorescence lifetime). Some proportion of the excited molecules will ultimately release their excess energy by emitting a photon as they drop back to the ground state. This creates fluorescence which can be separated from the excitation light via interference filters [5]. Volume images of the fluorescent yield and lifetime parameters are reconstructed from several optical measurements on the surface of the tissue [4]. 

Reconstruction of tomographic data from diffusing sources involves the generation of a forward model that predicts the photon distribution striking the detectors for a given source location and medium [6]. One challenging problem in the reconstruction process is that the computational complexity is very high due to an extremely large dimension of the matrix, which is not only in the inverse problem but also in the forward problem. Multiresolution approach is an effective way to speed up the process of solving the above problem. It is well known that the most important feature of the wavelet transforms lies in the fact that most information of the signal is contained in a small number of entries with other entries being very small and therefore can be neglected. In [7], an efficient pyramidal algorithm was proposed for the multiresolution representation of the signal with wavelets using orthogonal basis functions and quadrature mirror filters to compute it. Unser et al. extended these ideas to the case of nonorthogonal basis functions using splines [8]. Because the forward problem should be repeatedly solved during the process of solving the inverse problem especially for the case where there are large deviations in the optical properties between the target and the reference medium, the speed and accuracy of the forward computation are of critical importance determining the performance of the reconstruction algorithm. In order to speed up the forward computation process, we propose to generalize the strategy in [9] for solving the forward problem of FMT in the wavelet domain in combination with a parallel computing strategy [10]. Simulation results demonstrated that the proposed algorithm can significantly improve the efficiency of reconstruction for FMT. The main contribution of this paper is the extension of the multiresolution reconstruction approach originally developed for the diffuse optical tomographic reconstruction to the case of fluorescent molecular tomographic reconstruction suitable for the case where there are large deviations in the optical properties between the target and the reference medium. The forward problem of FMT is solved in wavelet domain in combination with a parallel computing strategy originally developed by our group, which decouple the two originally coupled differential equations corresponding to the excitation and the emission light, making the forward problem suitable for parallel implementation [10].

## 2. Forward Problem

### 2.1. Governing Equations

The generation and propagation of the excitation and fluorescence (emission) light in random highly scattering media can be described by two coupled diffusion equations which are the *P*_1_ approximation to the radiative transport equation (RTE). In the frequency domain, the diffusion equations become elliptic and can be expressed as

(1) $$-\nabla \cdot \left(D_{x}\nabla \Phi_{x}\right)+k_{x}\Phi_{x}=S_{x},$$

(2) $$-\nabla \cdot \left(D_{m}\nabla \Phi_{m}\right)+k_{m}\Phi_{m}=\beta \Phi_{x}$$

subjecting to the Robin boundary conditions on the boundary of the tissue

(3) $$\begin{array}{c} n\cdot \left[D_{x}\nabla \Phi_{x}\left(r\right)\right]+b_{x}\Phi_{x}\left(r\right)=0\;\forall r\in \partial \Omega ,\\ n\cdot \left[D_{m}\nabla \Phi_{m}\left(r\right)\right]+b_{m}\Phi_{m}\left(r\right)=0\;\forall r\in \partial \Omega , \end{array}$$

where (1) describes the propagation of the excitation light, and (2) models the generation and propagation of fluorescently emitted light. The subscripts *x* and *m* denote the excitation and emission light wavelengths, respectively. ∇ is the gradient operator, *S*_*x*_ is the excitation light source, and Φ_*x*,*m*_ is the photon fluence. **n** is a vector normal to the boundary ∂Ω, *b*_*x*_, and *b*_*m*_ are the Robin boundary coefficients which are governed by the reflection coefficients (*R*_*x*_,*R*_*m*_). The values of *b*_*x*_ and *b*_*m*_ are 1/2 (no reflection). In addition, the diffusion coefficients *D*_*x*,*m*_, decay coefficients *k*_*x*,*m*_, and emission source coefficients *β* are, respectively, defined as

(4) $$\begin{array}{cc} D_{x} & =\frac{1}{3\left(\mu_{axi}+\mu_{axf}+\mu_{sx}^{\prime }\right)},\\ D_{m} & =\frac{1}{3\left(\mu_{ami}+\mu_{amf}+\mu_{sm}^{\prime }\right)},\\ k_{x} & =\frac{i\omega }{c}+\mu_{axi}+\mu_{axf},\\ k_{m} & =\frac{i\omega }{c}+\mu_{ami}+\mu_{amf},\\ \beta & =\frac{\eta \mu_{axf}}{1-i\omega \tau }, \end{array}$$

where *μ*_*axi*_ (cm^−1^) and *μ*_*ami*_ (cm^−1^) are the absorption coefficients due to nonfluorescing chromophore; *μ*_*axf*_ (cm^−1^) and *μ*_*amf*_ (cm^−1^) are the absorption coefficients due to fluorophore; *μ*_*sx*_′ (cm^−1^) and *μ*_*sm*_′ (cm^−1^) are the isotropic scattering coefficients; *η* is the fluorescence quantum efficiency; *τ* (s) is the fluorescence lifetime; *c* (cm/s) is the speed of light in the media and $i=\sqrt{-1}$.

### 2.2. Finite Element Formulation

The solutions to (1) and (2) can be obtained using the finite element method (FEM) which is a completely general numerical technique applied to any geometry [11]. The FEM is one of the most popular methods for numerically solving partial differential equations (PDEs) because of its applicability to a range of problems and the existing large body of mathematical theory [12]. In the FEM framework, the domain is divided into *P* elements, joined at *N* vertex nodes. The solution Φ_*x*,*m*_ is approximated by the piecewise function Φ_*x*,*m*_ = ∑_*i*_^*N*^Φ_*xi*,*mi*_ *φ*_*i*_, with *φ*_*i*_ (*i* = 1,…, *N*) being basis functions [13]. 

Suppose *V*_0_^*h*^ = span{*φ*_*j*_}_*j* = 1_^*N*^ [14], ∀ *v*_*h*_ ∈ *V*_0_^*h*^, we have

(5) $$v_{h}=\overset{N}{\underset{k=1}{\sum }}c_{k}\varphi_{k}.$$

Let *u*_*h*_ = ∑_*j* = 1_^*N*^(Φ_*j*_)_*x*,*m*_ *φ*_*j*_, where *u*_*h*_ stands for both Φ_*x*_ and Φ_*m*_. In order to obtain the weak solutions of (1) and (2) under the boundary conditions of (3), (1) and (2) are written as the following sesquilinear form:

(6) $$a_{\Omega_{h}}{\left(u_{h},v_{h}\right)}_{x,m}={\left(f_{x,m},v_{h}\right)}_{\Omega_{h}},$$

where

(7) $$\begin{array}{cc} a_{\Omega_{h}}{\left(u_{h},v_{h}\right)}_{x,m} & =\iint_{\Omega_{h}}^{}\left[D_{x,m}\left(\nabla u_{h}\cdot \nabla v_{h}\right)+k_{k,m}u_{h}v_{h}\right]d\Omega +\int_{\Gamma_{h}}^{}b_{x,m}u_{h}v_{h}ds,\\ {\left(f_{x,m},v_{h}\right)}_{\Omega_{h}} & =\iint_{\Omega_{h}}^{}f_{x,m}v_{h}d\Omega ,\\ f_{x} & =S_{x},\\ f_{m} & =\beta \Phi_{x}, \end{array}$$

where Ω_*h*_ and Γ_*h*_ are, respectively, the bounded domain and its boundary. Equation (6) can be further rewritten in a more compact matrix form as

(8) $$A_{x}\Phi_{x}=S_{x},$$

(9) $$A_{m}\Phi_{m}=S_{m},$$

where

(10) $$\begin{array}{ll} S_{x,m} & =\left[\begin{array}{c} {\left(f_{x,m},\varphi_{1}\right)}_{\Omega_{h}}\\ \vdots \\ {\left(f_{x,m},\varphi_{N}\right)}_{\Omega_{h}} \end{array}\right],\\ A_{x,m} & =\left[\begin{array}{ccc} a_{\Omega_{h}}{\left(\varphi_{1},\varphi_{1}\right)}_{x,m} & \cdots & a_{\Omega_{h}}{\left(\varphi_{N},\varphi_{1}\right)}_{x,m}\\ \vdots & & \vdots \\ a_{\Omega_{h}}{\left(\varphi_{1},\varphi_{N}\right)}_{x,m} & \cdots & a_{\Omega_{h}}{\left(\varphi_{N},\varphi_{N}\right)}_{x,m} \end{array}\right]. \end{array}$$

The elements of finite element matrix **A**_*x*,*m*_ can be obtained from the following formula:

(11) $$a_{\Omega_{h}}{\left(\varphi_{i},\varphi_{j}\right)}_{x,m}=\iint_{\Omega_{h}}^{}D_{x,m}\nabla \varphi_{i}\cdot \nabla \varphi_{j}d\Omega +\iint_{\Omega_{h}}^{}k_{x,m}\varphi_{i}\varphi_{j}d\Omega +\int_{\Gamma_{h}}^{}b_{x,m}\varphi_{i}\varphi_{j}ds.$$

### 2.3. Forward Computation

The accuracy and speed of solving the forward problem as discussed in Section 1 are of critical importance determining the performance of the reconstruction algorithm. A multiresolution iterative perturbation reconstruction method for the optical tomographic image reconstruction based on the wavelet transform is presented in [9]. By computing the Jacobian matrix, which is a measure of the rate of changes in measurement with respect to the optical parameters, at a reference medium whose optical properties are similar to those of the target medium, the reconstruction problem is reduced to a system of linear equations. As a result, there is no need for repeated solving of the forward differential equations. However, this method would not be valid for the case where there are large deviations in the optical properties between the target and the reference medium. In such a case, the forward problem should be repeatedly solved during the process of solving the inverse problem. In order to speed up the forward computing process so as to speed up the whole process of reconstruction, we propose to generalize the strategy proposed in [9] originally developed for the inverse reconstruction of the diffuse optical tomography to the case of the forward problem of FMT and solve it in the wavelet domain. Furthermore, in order to decouple the forward problem of FMT, a parallel strategy previously developed by our group [10] will be used in combination with the aforementioned strategy for solving the forward problem. Our innovations are especially suitable for the case where there are large deviations in the optical properties between the target and the reference medium.

#### 2.3.1. Brief Introduction of Wavelets

For the convenience of the following discussion, a brief introduction of the theory of wavelet transform is presented here. The wavelet transform is a tool that cups up data of functions or operators into different frequency components and then studies each component with a resolution matched to its scale. By a proper design of the basis, the wavelet can project the signal onto a chain of embedded approximations and details at various levels of resolutions, and, as a result, the wavelet transform is usually referred to as the multiresolution analysis. For example, the two-level wavelet-based multiresolution representation of one dimensional discrete signal **f** with *N* components can be described as

(12) $${\tilde{f}}_{N\times 1}=\left[\begin{array}{c} A_{-1}f_{N/2\times 1}\\ D_{-1}f_{N/2\times 1} \end{array}\right],$$

where ${\tilde{f}}_{N\times 1}={\left[{\tilde{f}}_{1},{\tilde{f}}_{2},\ldots ,{\tilde{f}}_{N}\right]}^{T}$ is the wavelet transform of the original signal **f**. It can be seen from this equation that the original signal can be decomposed into two parts of the detail component *D*_−1_ **f**_*N*/2×1_ and the approximation component *A*_−1_ **f**_*N*/2×1_.

Similarly, the two-level wavelet-based multiresolution representation of a 2D image **F** sized *M* × *N* can be expressed with the following formula:

(13) $${\tilde{F}}_{M\times N}=\left[\begin{array}{cc} A_{-1}F_{M/2\times N/2} & D_{-1}^{1}F_{M/2\times N/2}\\ D_{-1}^{2}F_{M/2\times N/2} & D_{-1}^{3}F_{M/2\times N/2} \end{array}\right].$$

Four elements in the matrix of the right-hand side of (13) are, respectively, the approximation image *A*_−1_ **F**_*M*/2×*N*/2_ and three detail images *D*_−1_^1^ **F**_*M*/2×*N*/2_, *D*_−1_^2^ **F**_*M*/2×*N*/2_, and *D*_−1_^3^ **F**_*M*/2×*N*/2_.

#### 2.3.2. Multiresolution Computing of the Forward Problem for the Excitation Light in Wavelet Domain

In order to exploit the multiresolution property of the wavelet and reduce the forward computational time, the forward problem is first represented in the wavelet domain. For such a purpose, multiplying both sides of (8) from the left by **W**_**S**_ and assuming the orthonormality of **W**_Φ_, we have

(14) $${\tilde{A}}_{x}{\tilde{\Phi }}_{x}={\tilde{S}}_{x},$$

where ${\tilde{A}}_{x}=W_{S}A_{x}W_{\Phi }^{T}$, ${\tilde{S}}_{x}=W_{S}S_{x}$, ${\tilde{\Phi }}_{x}=W_{\Phi }\Phi_{x}$, **W**_**S**_ and **W**_Φ_ are, respectively, the wavelet transform matrix of **S** and Φ. 

It is well known that the most important feature of the wavelet transforms lies in the fact that most information of the signal is contained in a small number of entries with other entries being very small and therefore can be neglected. As a result, the dimension of the forward problem can be reduced level by level by using only the approximation components of the wavelet coefficients to describe the forward problem, that is,

(15) $${\tilde{A}}_{x,l}{\tilde{\Phi }}_{x,l}={\tilde{S}}_{x,l},\;l=-1,\ldots ,-L,$$

where *l* denotes the index of the scale, ${\tilde{A}}_{x,l}=W_{S,l}A_{x,l}W_{\Phi ,l}^{T}$, ${\tilde{S}}_{x,l}=W_{S,l}S_{x,l}$, ${\tilde{\Phi }}_{x,l}=W_{\Phi ,l}\Phi_{x,l}$, and **W**_**S**,*l*_ and **W**_Φ,*l*_ are, respectively, the wavelet transform matrix of **S**_*x*,*l*_ and Φ_*x*,*l*_ at the *l*th scale with **S**_*x*,*l*_ and Φ_*x*,*l*_ being, respectively, the approximation components of the corresponding signal at the (*l* + 1)th scale, and **A**_*x*,*l*_ is the LL components of the corresponding wavelet transformed stiffness matrix at the (*l* + 1)th scale. 

Using the above multiresolution representation, the forward problem can be solved in a fine-to-coarse-to-fine procedure which can be summarized as in Algorithm 1.

Owing to the fact that some important features are contained in the coarse resolution solution, as a result, it will be very helpful for speeding up the iterative process when solving the forward problem at a higher level resolution with the solution obtained at a coarser resolution as an initial guess. Therefore, we can expect to expedite the process of solving the forward problem by using Algorithm 1  with a fine-to-coarse-to-fine strategy.

#### 2.3.3. Parallel Implementation of the Forward Problem for the Emission Light

After the discussion of the wavelet-based algorithm for the forward problem corresponding to the excitation light, the next task for us will be that of solving the forward problem for the emission photons. For the case where there are large deviations between the referenced and target medium, the forward equations must be solved repeatedly during the process of reconstruction following a model-based iterative image reconstruction scheme. Therefore, a rapid and accurate computational implementation of the forward problem is of critical importance for fluorescent molecular tomographic image reconstruction. From (8) and (9), we can see that the two forward models corresponding to the excitation and emission light at different wavelength coupled together because the solution to (8) is contained in the source term of (9). Traditionally, the forward problem of (8) and (9) are solved in a sequential manner, that is, (8) is first solved whose solution is then substituted to (9), which yields the photon fluence at the emission wavelength. That scheme will affect the computational speed of the forward problem, even the inverse problem. To tackle such a problem, an approximate computing strategy for decoupling these two forward equations was proposed in [15] and was used for the FMT reconstruction in [16]. However, this strategy is not valid for the case where there is a large stokes shift [15]. For a rapid implementation of the forward problem, we have proposed an accurate parallel implementation scheme in [10] where the following equation is solved instead of (9):

(16) $$\;A_{m}H=I.$$

In (16), **I** is an identity matrix. Since **A**_*x*,*m*_ is symmetric and positive definite [10], we can always obtain an inverse matrix **H** for **A**_*m*_ from (16). The matrix **H** can be obtained with the numerical method which can be speeded up when the matrix is symmetric and positive definite [17]. Combining (8) and (16) leads to a system of equations in discretized domain for the forward problem of FMT. Because (8) and (16) are independent, they can be solved in a parallel manner. Obviously, the photon fluence of Φ_*m*_ at the emission wavelength can be recovered by simple matrix multiplication with Φ_*x*_ contained in **S**_*m*_ obtained from Algorithm 1, that is,

(17) $$\Phi_{m}=H\cdot S_{m}.$$

In summary, the whole forward computation process in our proposed algorithm can be realized with Algorithm 1  in the wavelet domain for the excitation light and in a parallel manner for the emission light. It has been proved in our simulations that our proposed forward computing algorithm can significantly reduce the computational requirements.

#### 2.3.4. Computational Complexity Analysis of the Parallel Computing Strategy

The most important aspect of the parallel computing strategy is decoupling of the two coupled equations. In order to illustrate the improvement of the parallel computing strategy in computational complexity as compared with the sequential one quantitatively, the computing efficiency is analyzed as follows.

Because the maximum computational complexity of solving linear equations defined by a matrix sized *N* × *N* with CGD method is *O*(*N* ^3^) (the maximum iteration number for such an optimization problem is *N* [18], and the complexity of each iteration is *O*(*N* ^2^)) [19], the complexity of solving the coupled forward problem of FMT, that is, (8) and (9), in a sequential manner will be *O*(*N* ^3^) + *O*(*N* ^3^). On the other hand, if the forward problem is solved according to the parallel strategy as discussed previously, the computational complexity will be *O*(*N* ^3^) + *O*(*N* ^2^) because the operations of matrix inversion in (16) and solving the linear equation in (8) can be implemented independently with two processors simultaneously whose computational complexities are *O*(*N* ^3^), while the computational complexity of multiplying the matrix **A**_*x*,*m*_ by a vector in (17) is *O*(*N* ^2^) [20]. Thus, the speed of forward computing can be improved in such a parallel manner. The above analysis is valid for both two-dimensional (2D) and three-dimensional (3D) cases, because the only difference between these two cases is that the size *N* of the matrix **A**_*x*,*m*_ for 3D case is much larger than that of 2D case. 

Particularly, if we are interested only in the reconstruction of the absorption coefficient *μ*_*axf*_ due to the fluorophore, the matrix **H** in (16) needs to be calculated only once during the whole reconstruction process and hence the computational requirements are extremely reduced.

## 3. Image Reconstruction of FMT

### 3.1. Inverse Problem

The forward and inverse problem of FMT can be, respectively, formulated as

(18) $$\begin{array}{c} y=F\left(x\right),\\ x=F^{-1}\left(y\right), \end{array}$$

where *y* is the detector readings, *F* is the forward operator, and *x* is the optical or fluorescent properties of the tissue.

Generally, *y* is a nonlinear function of *x*. In order to simplify the reconstruction process, we expand the function *F* in the vicinity of *x*_0_ in a Taylor series [21]:

(19) $$y=y_{0}+F^{\prime }\left(x_{0}\right)\left(x-x_{0}\right)+\frac{1}{2}F^{\prime \prime }\left(x_{0}\right){\left(x-x_{0}\right)}^{2}+\cdots ,$$

where *F*′ and *F*′′ are the first- and second-order Frechet derivatives of *F* and are usually referred to as the Jacobian matrix and Hessian matrix, respectively, if represented in matrix form. Keeping up to the first-order terms in (19) and introducing the Tikhonov regularization term for tackling the ill-posedness of the inverse problem, the linearized formulation for the reconstruction problem can be described by

(20) $$x-x_{0}={\left(J^{T}J+\lambda I\right)}^{-1}J^{T}\left(y-y_{0}\right),$$

where **I** is an identity matrix, *λ* is a regularization parameter, and **J** is the Jacobian matrix describing the influence of a voxel on a detector reading *y* [22]. The Jacobian matrix is obtained using the perturbation method which can be described as

(21) $$J=\frac{\partial F\left(x\right)}{\partial x}\approx \frac{F\left(x+\Delta x\right)-F\left(x\right)}{\Delta x},$$

where Δ*x* is the perturbation in the optical or fluorescent properties, and *F*(*x* + Δ*x*) − *F*(*x*) stands for the corresponding residual data between the two predicted data.

By introducing two quantities of Δ**x** and Δ**y** which are, respectively, the perturbation in the optical or fluorescent properties and residual data between the measurements and the predicted data, (20) can be rewritten in a more compact matrix form as [23]

(22) $$\;\begin{array}{c} K\Delta x=J^{T}\Delta y=b \end{array}$$

with **K** = (**J** ^*T*^ **J** + *λ* **I**) and **b** = **J** ^*T*^Δ**y**.

Using (22), we can obtain the reconstructed image simply by finding a solution of Δ**x** to it. In our case, both the matrix **K** and the vector **b** and hence the Jacobian matrix **J** and the residual data Δ**y** are functions of Δ**x** considering the fact that there are large deviations between the target and reference medium, which is different from the case in [9] where **K** and **J** are irrelative to Δ**x** and remain unchanged during the iteration process. Therefore, both **J** and Δ**y** should be repeatedly calculated during the reconstruction process if iterative method is used to find a solution to (22), which means that the forward problem should also be repeatedly solved in the reconstruction process. As a result, the reconstruction efficiency can be significantly improved if we can expedite the repeated forward computation. As mentioned before, we can expect to expedite the process of the forward computing with an algorithm in the wavelet domain discussed in Section 2. In order to further speed up the reconstruction algorithm, we propose to adopt the multiresolution reconstruction scheme in the inversion process. For such a purpose, we perform the wavelet transform on both sides of (22) and have

(23) $$\tilde{K}\Delta \tilde{x}=\tilde{b,}$$

where $\tilde{K}=W_{b}KW_{x}^{T}$, $\Delta \tilde{x}=W_{x}\Delta x$, $\tilde{b}=W_{b}b$, **W**_**x**_ and **W**_**b**_ are, respectively, the wavelet transform matrix of Δ**x** and **b**, and **W**_**x**_ is an orthonormal matrix. As for the case of the forward problem, the wavelet transform can be successively performed level by level with respect to the approximation components of both sides of (23) and obtain a multiresolution representation of the reconstruction problem. The whole reconstruction algorithm can be summarized as in Algorithm 2.

From Algorithm 2, we can see that there are actually two layers of iterations in it: one is the inner iteration where the Jacobian matrix is not updated which is similar to that proposed in [9], and the other is the outer iteration where both the Jacobian matrix and the residual vector are recomputed at the new values of the optical parameters obtained in the former iteration. Owing to the fact that both the Jacobian matrix and the residual vector are updated during the outer iterations, our algorithm can free the constraints of small deviations of the properties between the target and the reference medium.

### 3.2. Data Correction

Actually the fluorescent measurements are used as the input to reconstruct the image for FMT according to Section 2. Usually the fluorescence may exist not only in the target but also in the background [24]. When the fluorescence image is reconstructed, it may contain the target fluorescence as well as the background fluorescence. Therefore, if the detector readings are directly used for image reconstruction, the performance of the reconstruction result will drop [25]. In order to improve the reconstruction quality in the presence of the background fluorescence, the data of reconstruction need to be corrected.

In the presence of the background fluorescence, the fluorescence concentration can be formulated as follows [25]:

(24) $$n=n_{\text{target}}+n_{\text{back}},$$

where *n* is the total fluorescence concentration, and *n*_target_ and *n*_back_ denote the target and background fluorescence concentration, respectively.

Furthermore, the fluorescence concentration can be described as follows [25]:

(25) $$n=\eta \mu_{a},$$

where *η* is the fluorescence quantum efficiency, and *μ*_*a*_ is the absorption coefficients.

According to (24) and (25), the absorption coefficients of the fluorescence can be formulated as the sum of the absorption coefficients of the target and background fluorescence, that is,

(26) $$\mu_{a}=\mu_{a,\text{target}}+\mu_{a,\text{back}},$$

where *μ*_*a*,target_ and *μ*_*a*,back_ are the absorption coefficients of the target and background fluorescence, respectively. 

In order to improve the reconstruction quality in the presence of the background fluorescence, the reconstruction results can be corrected as follows [25]:

(27) $$\mu_{\text{a},\text{corrected}}=\mu_{a}-\frac{1}{S_{\Omega_{h}}}\iint_{\Omega_{h}}^{}\mu_{a}d\Omega ,$$

where Ω_*h*_ is the domain of interest for the reconstruction with an area of *S*_Ω_*h*__. From (27), it can be seen that *μ*_*a*,back_ can be obtained by taking the average of the reconstructed absorption coefficients especially when the variations of background fluorescence concentration are small. Additionally, as no negative fluorescence exits, any negative value of *μ*_a,corrected_ should be set to zero.

## 4. Simulation Results

### 4.1. Two-Dimensional Reconstruction

**Figure 1:**
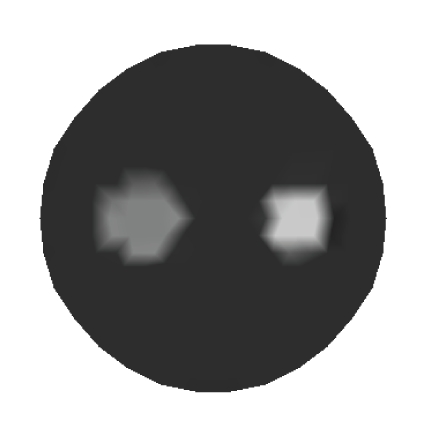
Model of reconstruction.

The algorithm proposed in this paper has been firstly tested in a 2D simulated phantom with two anomalies existing within it as illustrated in Figure 1. Four sources and thirty detectors equally distributed around the circumferences of the phantom are adopted in the simulations. The optical and fluorescent parameters in different regions of simulated phantom are listed in Table 1. The simulated forward data are obtained from (1) and (2), in which the Gaussian noise with a Signal-to-Noise Ratio of 15 dB is added for evaluating the noise robustness of the algorithms. Furthermore, the background fluorescence is also included in the simulated data for evaluating the data correction strategy according to Table 1. Since Daubechies 1 (haar wavelet) has the advantages such as orthogonality and symmetry, Daubechies 1 wavelet as defined in (28) is used in the simulations [26]:

(28) $$\psi \left(t\right)=\{\begin{array}{ll} 1, & 0\leq t<\frac{1}{2},\\ -1, & \frac{1}{2}\leq t<1,\\ 0, & \text{others}. \end{array}$$

**Figure 2:**
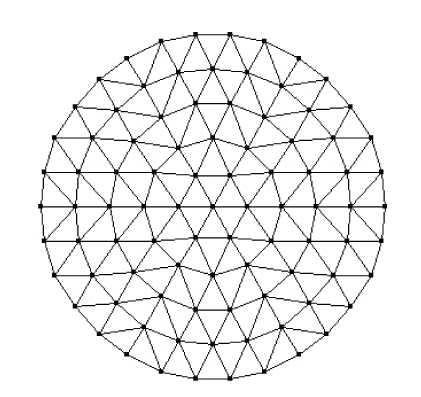
Uniform mesh.

In our current implementation, we will focus on reconstructing the distributions of absorption coefficients *μ*_*axf*_ due to the fluorophore. The termination criterion *ε* in Algorithm 2  is set to 0.02. The regularization parameter *λ* is set to 0.005 in the simulations for better results after a lot of simulations [27]. The uniform mesh for reconstruction is shown in Figure 2 with 91 vertex nodes in it. Two quantities are introduced for the quantitative evaluations of different algorithms. The first one is the error function *E* between the simulated data and the predicted data computed at the final reconstructed value, that is,

(29) $$E=\frac{1}{N}{\left\|F(x)-y\right\|}^{2},$$

where *N* is the number of vertex nodes, **y** is the detector readings, *F* is the forward operator, **x** is the reconstructed result of optical or fluorescent properties of tissue, and ‖·‖^2^ is *L*_2_-norm. The second one is the normalized root mean squares (NRMSs) error for the reconstructed results defined as

(30) $$\;\text{NRMS}={\left\{\frac{\sum_{i=1}^{N}{\left({\tilde{x}}_{i}-x_{i}\right)}^{2}}{\sum_{i=1}^{N}{\left({\tilde{x}}_{i}-{\overset{̅}{x}}_{i}\right)}^{2}}\right\}}^{1/2},$$

where *N* is the number of vertex nodes, ${\tilde{x}}_{i}$ and *x*_*i*_ are the original pixel and reconstructed pixel values, respectively, and ${\overset{̅}{x}}_{i}$ is the mean value of the original pixel.

**Figure 3:**
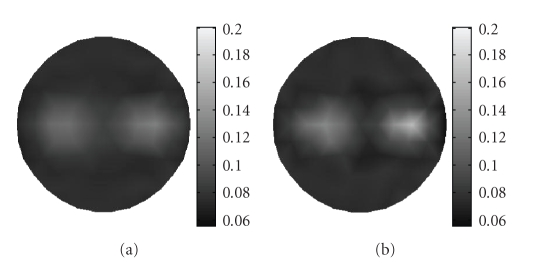
Reconstructed image of absorption coefficient due to fluorophore *μ*_*axf*_ (a) without data correction, and (b) with data correction.

The data correction strategy is implemented after the reconstruction for improving the reconstruction quality. Figure 3(a) depicts the reconstructed result of absorption coefficients *μ*_*axf*_ without data correction, and Figure 3(b) shows the corresponding result after data correction, both of them are based on the proposed wavelet-based multiresolution algorithm. From these two images, it can be seen that the data correction strategy can improve the image quality. Hence, all reconstruction results presented in the latter part of this section are those after data correction.

**Figure 4:**
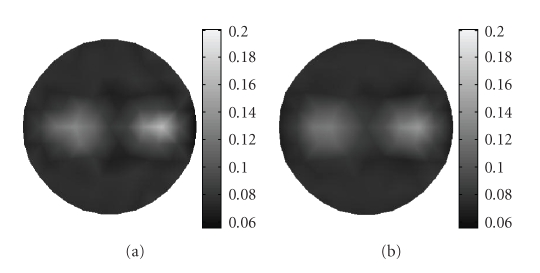
Reconstructed image of absorption coefficient due to fluorophore *μ*_*axf*_ with (a) proposed algorithm, and (b) method in [9].

Figures 4(a)  and 4(b)  show the reconstructed images of *μ*_*axf*_ using the proposed algorithm and the method in [9], respectively. In this example, the deviation of the optical properties between the reference and the target medium is set to a relatively larger value (here **x**_0_ is set to 10 mm^−1^) for an illustration of the reliabilities of the above two different algorithms under the circumstance with large deviations. These two algorithms are implemented with a same initial guess of 10. Table 2 summarizes the performance of these two algorithms in terms of *E* and NRMS. From this table it can be seen that the proposed algorithm can achieve a more accurate reconstructed result for the case where there are large deviations in the optical properties between the target and the reference medium, that is, our algorithm is more suitable for such a case.

**Figure 5:**
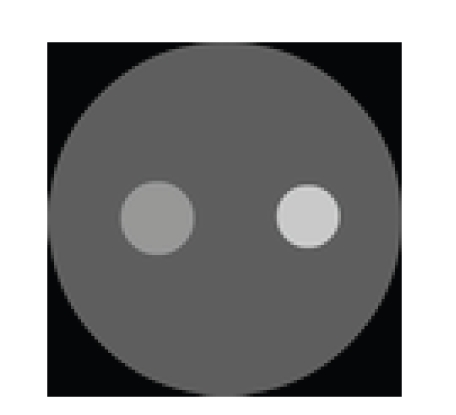
Model of prior image.

**Figure 6:**
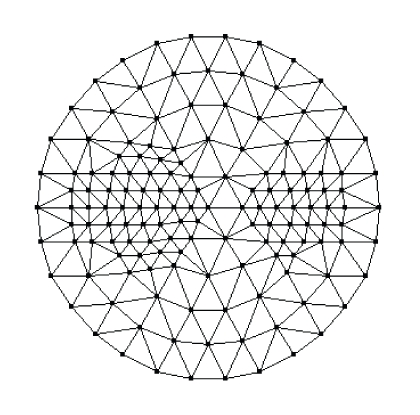
Adaptively refined mesh.

Recently, there has been a great amount of interest in developing multimodality imaging techniques for oncologic research and clinical studies with the aim of obtaining complementary information and, thus, improving the detection and characterization of tumors [28]. As a result, it will be helpful to incorporate the prior information obtained from other imaging modalities in the reconstruction process for reducing the computational requirements while achieving a relatively better reconstructed result. In our case, we proposed to use the prior information to generate a nonuniform mesh for the fluorescent image reconstruction according to an adaptively refinement scheme. The basic idea of this scheme is that, for areas in the prior image with large variations of the pixel values, the mesh at this position should be locally refined, and hence the image will be reconstructed with higher resolutions, whereas for regions with small variations, the mesh at this position should be left unchanged, and hence the image will be reconstructed with low resolutions locally at this position correspondingly. Obviously, this idea is plausible because flat regions contain little information and therefore low resolution reconstruction will not lead to serious degradation of the reconstructed results. However, this nonuniform reconstruction will significantly reduce the computational requirements as compared with the uniform fine reconstruction. To simulate such an idea in fluorescent image reconstruction, we use the image shown in Figure 5 with a resolution of 100 × 100 pixels as a prior image. Figure 6 shows the adaptively refined mesh with 148 vertex nodes in it which is generated based on the above idea. All of the following reconstructed results of this section are obtained based on this adaptively refined mesh.

**Figure 7:**
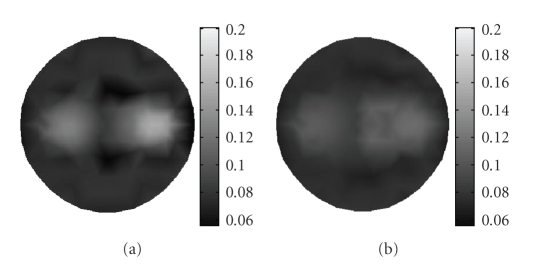
Reconstructed image of absorption coefficient due to fluorophore *μ*_*axf*_ based on adaptively refined mesh with (a) wavelet-based algorithm, and (b) single resolution method.

Furthermore, in order to demonstrate the advantage of the proposed algorithm as compared with the traditional single resolution method without wavelet transform, Figures 7(a)  and 7(b)  depict the reconstructed images using the proposed wavelet-based multiresolution reconstruction algorithm and the single resolution method, respectively, both of which are implemented in combination with the parallel forward computing strategy for further speeding up the process of the reconstruction. Table 3 summarizes the performance of the different algorithms in terms of the computation time, NRMS and *E*, from which we can see that our wavelet-based algorithm outperforms the single resolution algorithm in both the reconstruction accuracy and the computational requirements. 

### 4.2. Three-Dimensional Reconstruction

To further validate the proposed algorithm for 3D reconstruction, we extend the methods previously defined for triangular elements to tetrahedral elements. Therefore, the shape functions in the local coordinate system (*x*′, *y*′, *z*′) is defined as

(31) $$\begin{array}{c} L_{1}=\frac{1}{2}\left(1-x^{\prime }-\frac{y^{\prime }}{\sqrt{3}}-\frac{z^{\prime }}{\sqrt{6}}\right),\\ L_{2}=\frac{1}{2}\left(1+x^{\prime }-\frac{y^{\prime }}{\sqrt{3}}-\frac{z^{\prime }}{\sqrt{6}}\right),\\ L_{3}=\frac{y^{\prime }}{\sqrt{3}}-\frac{z^{\prime }}{2\sqrt{6}},\\ L_{4}=\frac{1}{2}\sqrt{\frac{3}{2}}z^{\prime }. \end{array}$$

The integration of products of shape functions over the volume of the elements, and surface integrals over a side of the element, as required for the computation of element stiffness and mass matrices, is performed by a numerical integration rules. Once the element matrices are computed, the FEM model can be solved as in the 2D case without needs for any further alteration.

**Figure 8:**
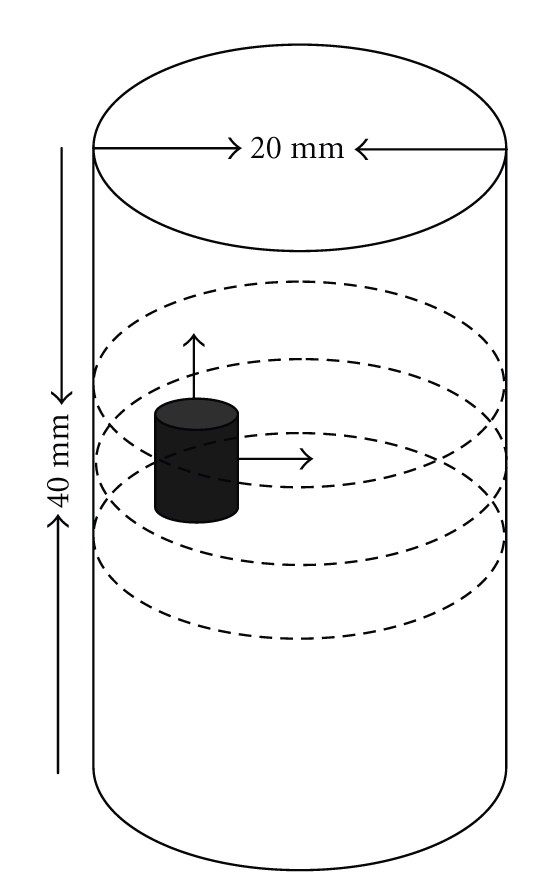
Schematic diagram of the phantom of radius 10 mm and height 40 mm with a uniform background of *μ*_*axf*_ = 0.005 mm^−1^, which is positioned at *x* = 10 mm, *y* = 0 mm, and *z* = 20 mm. The small cylindrical anomaly has a radius of 2 mm and height 6 mm with *μ*_*axf*_ = 0.01 mm^−1^. The anomaly is positioned at *x* = 5 mm, *y* = 0 mm, and *z* = 20 mm. The dashed curves represent the measurement planes, at *z* = 15 mm, *z* = 20 mm, *z* = 25 mm, each containing four sources and sixteen measurements.

**Figure 9:**
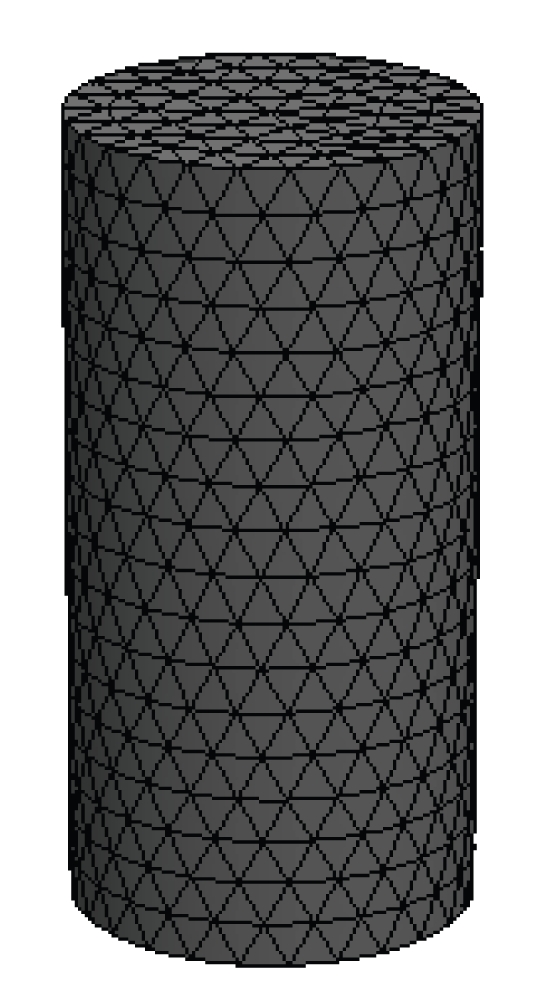
3D mesh for image reconstruction with 858 nodes and 3208 tetrahedral elements.

**Figure 10:**
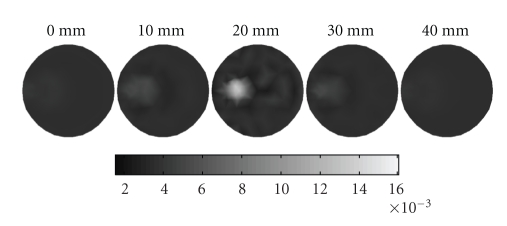
Reconstructed images using the proposed algorithm, which are 2D cross sections through the reconstructed 3D volume. The right-hand side corresponds to the top of the cylinder (*z* = 40 mm), whereas the left corresponds to the bottom of the cylinder (*z* = 0 mm), with each slice representing a 10 mm increment.

**Figure 11:**
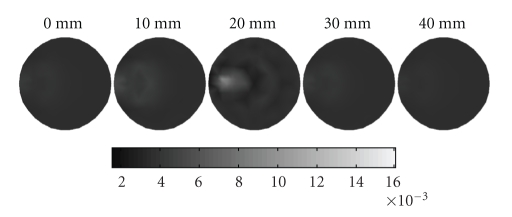
Reconstructed images using the single resolution method, which are 2D cross sections through the reconstructed 3D volume. The right-hand side corresponds to the top of the cylinder (*z* = 40 mm), whereas the left corresponds to the bottom of the cylinder (*z* = 0 mm), with each slice representing a 10 mm increment.

In the 3D case, a phantom of radius 10 mm and height 40 mm with a uniform background *μ*_*axf*_ = 0.002 mm^−1^ as illustrated schematically in Figure 8 is used for simulations. Within this phantom, a small cylindrical object of radius 2 mm and height 6 mm with *μ*_*axf*_ = 0.016 mm^−1^ is suspended. In Figure 8, the dashed curves represent the planes of measurement, 5 mm apart with *z* coordinates of 15, 20, and 25 mm*.* Four sources and sixteen measurements are used for each plane in the simulations. The mesh for reconstructing the 3D image as shown in Figure 9 is a cylindrical mesh of radius of 10 mm and height 40 mm. It contains 858 nodes and 3208 tetrahedral elements. The data are collected in all three measurement planes, as shown in Figure 8. Figures 10  and 11  depict the 3D reconstructed images using the proposed algorithm and the single resolution method, respectively. These are 2D cross sections through the reconstructed 3D images. The right-hand side corresponds to the top of the cylinder (*z* = 40 mm), and the left corresponds to the bottom of the cylinder (*z* = 0 mm), with each slice corresponding to a 10 mm increment in the *z* coordinates. 

Table 4 lists the performance of the above two methods for a quantitative comparison in detail. It can be seen that the proposed algorithm can significantly speed up the process of reconstruction and improve the reconstructed image quality. Therefore we can conclude that our proposed algorithm also outperforms the single resolution reconstruction algorithm without wavelet transform for the 3D case. 

Furthermore, the proposed algorithm decouples the two coupled equations for the forward problem of FMT, and thus it is quite suitable for parallel computing of the two independent equations with two processors. Table 5 summarizes the computation time and rate of speedup with different number of processors used to validate the superiority of the proposed parallel computing strategy. From Table 5, it can be seen that the parallel computing strategy can speed up the reconstruction process both in the 2D and 3D reconstruction. The rate of speedup for the 3D case is a little higher than that for the 2D case, which indicates that the superiority of parallel computing strategy is more prominent in 3D reconstruction than in 2D case.

## 5. Conclusion

In summary, a wavelet-based multiresolution reconstruction algorithm is proposed in combination with the parallel forward computation strategy for the purpose of speeding up the reconstruction process with an improved reconstruction accuracy. The most important contribution of this paper is the novel extension of the multiresolution reconstruction approach originally developed for the diffuse optical tomographic reconstruction to the case of fluorescent molecular tomographic reconstruction and for the case where there are large deviations of the optical parameters between the target and the reference medium. Different from the algorithm proposed in [9], the forward problem of FMT is solved in wavelet domain in combination with a parallel computing strategy for speeding up the forward computing process which is especially suitable for the case where there are large deviations in the optical properties between the target and the reference medium, and thus the forward problem should be computed repeatedly. Simulation results demonstrate that the proposed algorithm can significantly reduce the computational complexity and achieve a higher reconstruction quality.

## Figures and Tables

**Algorithm 1:**
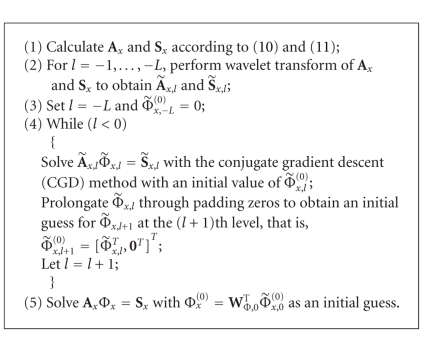


**Algorithm 2:**
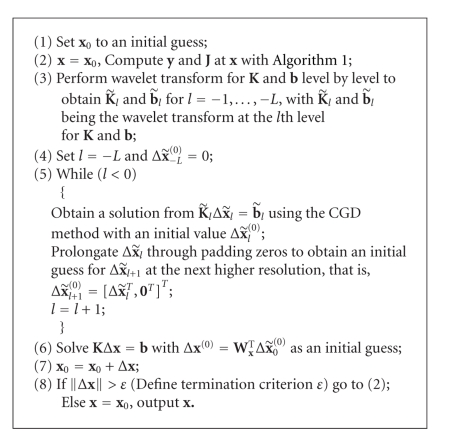


**Table 1:** Optical and fluorescent properties.

Excitation light	*μ*_*axi*_ (mm^−1^)	*μ*_*axf*_ (mm^−1^)	*μ*_*sx*_′ (mm^−1^)	*η*	*τ* (ns)	*μ*_*axi*_ (mm^−1^)
Background	0.06	0.06	5.0	0.3	0.5	0.06
Anomalies	0.06	0.15, 0.2	5.0	0.3	0.5	0.06

Fluorescent light	*μ*_*ami*_ (mm^−1^)	*μ*_*amf*_ (mm^−1^)	*μ*_*sm*_′ (mm^−1^)	*η*	*τ*(ns)	*μ*_*ami*_ (mm^−1^)

Background	0.02	0.006	2.0	0.3	0.5	0.02
Anomalies	0.02	0.05, 0.1	2.0	0.3	0.5	0.02

**Table 2:** Performance comparison of reconstruction algorithms.

Performance	Reconstruction without data correction	Method in [9]	Proposed algorithm
*E*	1.114 × 10^−7^	6.573 × 10^−8^	2.312 × 10^−8^
NRMS	6.798 ×10^−3^	5.238 × 10^−3^	3.471 × 10^−3^

**Table 3:** Performance comparison of reconstruction algorithms.

Performance	Single resolution method	Proposed multiresolution algorithm
Computation time (s)	247	185
NRMS	6.623 × 10^−3^	2.679 × 10^−3^
*E*	7.985 × 10^−8^	1.572 × 10^−8^

**Table 4:** Performance comparison of reconstruction methods.

Performance	Single resolution method	Proposed multiresolution algorithm
Computation time (s)	3196	2279
NRMS	9.114 × 10^−2^	2.043 × 10^−2^
*E*	1.963 × 10^−5^	4.566 × 10^−6^

**Table 5:** Efficiency analysis of parallel computing strategy.

	2D case		3D case	
Number of processors	1	2	1	2
Computation time (s)	292	185	3712	2279
Speedup	1.00	1.58	1.00	1.63
